# Monocyte Activation in HIV/HCV Coinfection Correlates with Cognitive Impairment

**DOI:** 10.1371/journal.pone.0055776

**Published:** 2013-02-21

**Authors:** Hans Rempel, Bing Sun, Cyrus Calosing, Linda Abadjian, Alexander Monto, Lynn Pulliam

**Affiliations:** 1 Department of Laboratory Medicine, Veterans Affairs Medical Center, San Francisco, California, United States of America; 2 Department of Mental Health, Veterans Affairs Medical Center, San Francisco, California, United States of America; 3 Department of Medicine, Veterans Affairs Medical Center San Francisco, California, United States of America; 4 University of California San Francisco, San Francisco, California, United States of America; Imperial College London, United Kingdom

## Abstract

Coinfection with human immunodeficiency virus (HIV) and hepatitis C virus (HCV) challenges the immune system with two viruses that elicit distinct immune responses. Chronic immune activation is a hallmark of HIV infection and an accurate indicator of disease progression. Suppressing HIV viremia by antiretroviral therapy (ART) effectively prolongs life and significantly improves immune function. HIV/HCV coinfected individuals have peripheral immune activation despite effective ART control of HIV viral load. Here we examined freshly isolated CD14 monocytes for gene expression using high-density cDNA microarrays and analyzed T cell subsets, CD4 and CD8, by flow cytometry to characterize immune activation in monoinfected HCV and HIV, and HIV-suppressed coinfected subjects. To determine the impact of coinfection on cognition, subjects were evaluated in 7 domains for neuropsychological performance, which were summarized as a global deficit score (GDS). Monocyte gene expression analysis in HIV-suppressed coinfected subjects identified 43 genes that were elevated greater than 2.5 fold. Correlative analysis of subjects’ GDS and gene expression found eight genes with significance after adjusting for multiple comparisons. Correlative expression of six genes was confirmed by qPCR, five of which were categorized as type 1 IFN response genes. Global deficit scores were not related to plasma lipopolysaccharide levels. In the T cell compartment, coinfection significantly increased expression of activation markers CD38 and HLADR on both CD4 and CD8 T cells but did not correlate with GDS. These findings indicate that coinfection is associated with a type 1 IFN monocyte activation profile which was further found to correlate with cognitive impairment, even in subjects with controlled HIV infection. HIV-suppressed coinfected subjects with controlled HIV viral load experiencing immune activation could benefit significantly from successful anti-HCV therapy and may be considered as preferential candidates.

## Introduction

Approximately 25% of individuals infected with human immunodeficiency virus (HIV) are coinfected with hepatitis C virus (HCV) in the US and Western Europe and 95% of injection drug users with HIV are coinfected with HCV [1]. Coinfection is associated with rapid progression of liver fibrosis, poor response to pegylated interferon (peg-IFN) and ribavirin, and increased incidence of cognitive impairment [2], [3], [4]. HIV monoinfection has long been associated with severe cognitive impairment and dementia toward the end stage of the illness. Following the introduction of antiretroviral therapy (ART), severity of neuropsychological impairment diminished dramatically but a substantial portion of patients continued to suffer from mild cognitive problems (for review [5]). While the neurologic impact of HCV monoinfection has been less pronounced, subtle impairment has been detected in patients who are carefully screened to avoid cofounding factors [6]. HCV infection has also been found to affect brain metabolites with monoinfected patients displaying a decrease in the N-acetylaspartate/creatine ratio in the cerebral cortex [7]. Yet, even in the context of HIV and HCV monoinfection, coinfection results in a statistically significant increase in cognitive compromise compared to each virus separately [8], [9], [10].

In HIV disease, peripheral immune activation and specifically T cell activation is a valid indicator of disease progression, exceeding even viral load as a prognostic marker [11], [12]. Immune activation, which is generally related to HIV viral load, is dramatically diminished when effective ART suppresses viral replication. It has been reported that CD8 T cell markers CD38 and HLADR were increased in HIV/HCV coinfected women, but not in HIV-monoinfected women with suppressed viral loads [13]. Elevated CD38 expression was also detected in both CD4 and CD8 T cell subsets of coinfected men and women compared to monoinfected individuals, which was subsequently lowered in individuals who achieved sustained HCV viral repression following peg-IFN/ribavirin treatment [14]. Immune activation was also detected in gene expression profiles of peripheral blood mononuclear cells (PBMCs) from coinfected individuals using cDNA microarray analysis [15]. Comparison with gene expression profiles from HIV-infected individuals with viremia revealed an aberrant type 1 IFN response in the coinfected [15].

While cognitive impairment and a type 1 IFN PBMC activation have been characterized separately in coinfected individuals, these occurrences have not been studied concurrently in the same cohort. We have previously reported that subjects chronically infected with HIV have a monocyte type 1 IFN gene expression profile that correlates with viral load [16], [17]. Here, in this study, we add analyses of monocyte gene expression in HCV monoinfected and HIV/HCV coinfected subjects using the same high-density cDNA microarray platform and analysis. One study objective was to determine how coinfection, where all subjects were on ART with HIV viral loads below the detectable levels, impacted monocyte gene expression as compared to HCV and HIV monoinfections. Having determined that coinfected individuals in this cohort had a higher prevalence of cognitive impairment [18], our second aim was to identify genes in the monocyte activation profile that correlated with the individual’s global deficit score (GDS). This type of analysis might pinpoint peripheral immune responses linked to neurocognitive dysfunction. Finally, we examined whether CD4 and CD8 T cell activation markers, CD38 and HLADR, were associated with lower cognition in the HIV-suppressed coinfected.

## Materials and Methods

### Subjects

Individuals were recruited from the San Francisco Veterans Affairs Medical Center (SFVAMC). HIV- and HCV-infected subjects were clinically stable outpatients while controls were healthy individuals associated with or receiving preventative care at the SFVAMC. This was a cross-sectional study where subjects provided written consent to participate in a research protocol that was approved by the University of California, San Francisco Committee on Human Research. Subjects were male, 45 to 65 years of age with either HCV monoinfection or HIV/HCV coinfection along with healthy controls. Subjects with HCV were viremic and infected with genotype 1. Coinfected subjects were compliant on ART with undetectable HIV viral load (<50 copies/ml) for 6 months prior to enrollment. Exclusion criteria for participating individuals included: ongoing illicit drug use by self-report, prescribed opiates or other psychoactive medications 6 months prior to enrollment, clinical evidence of cirrhosis or chronic infections other than HCV or HIV documented by liver biopsies, alcohol consumption >20 grams per day, IFNα-based therapy within the previous four years, clinical depression or other significant psychiatric disease, seizure disorder or history of head injury.

In addition to the HCV cohort described in this study, we included data obtained from an HIV cohort that was previously published [17], [19]. For the HIV cohort, monoinfected subjects were on ART or drug holiday and were subdivided into 2 groups based on viral load: undetectable HIV (HIV_UD_; n = 14; <50 copies/ml) and HIV (n = 22; ≥10,000 copies/ml). Also included were HIV seronegative healthy controls (n = 11). Subjects were recruited at the SFVAMC, receiving stable medical care with the same exclusion criteria as the HCV and HIV-suppressed coinfected subjects. Monocyte gene expression analysis in the HIV cohort used the same high-density microarray platform and computational tools as that for the HCV cohort and the expression data has been deposited in the public database (NCBI GEO database, accession number GSE18464). HIV and HCV cohorts were evaluated using identical neuropsychological tests conducted by the same clinical psychologist (L.A.).

### cDNA Microarray Analysis

Whole blood was collected in Vacutainer CPT tubes from Beckton Dickinson Biosciences (BD) and PBMCs were enriched by centrifugation. From 30 ml of whole blood, ∼3×10^6^ CD14 monocytes were isolated by immunomagnetic positive selection using anti-CD14 monoclonal antibodies conjugated to ferrous beads per manufacturer’s guidelines (Miltenyi Biotech). Monocyte purity exceeded 97% with <1% T or B cell contamination as determined by flow cytometry. Monocyte RNA was isolated with Qiagen RNeasy Micro Kit having an RNA integrity value >9 [20]. Complementary DNA was synthesized and labeled with biotin (iExpress iAmplify kit, Applied Microarrays) and hybridized to Codelink Whole Human Genome Bioarrays (55 K probes, Applied Microarrays). Slides were scanned (Axon GenePix 4000B, Molecular Devices), analyzed (CodeLink Expression Software Kit v4.1) and microarray data were normalized with loess normalization using R [21] and Bioconductor packages [22].

### PCR Analysis

For each gene, primers were designed using Primer-BLAST software (http://www.ncbi.nlm.nih.gov/tools/primer-blast/) for amplicons in the range of 100–200 bp with primer melting temperatures of 58–62C. Primer sets spanned exon junctions or intron inclusions. Primer sets were as follows: GAPDH (NM_002046) forward: 5′- ATTCCACCCATGGCAAATTC-3′, reverse 5′- TGGGATTTCCATTGATGACAAG-3′; HES4 forward: 5′-ACCCTCATCCTGGACGCCCTC-3′, reverse 5′-TACTTGCCCAGAACGGCGGG-3′; IFI27 forward: 5′- TGGCCTCTGGCTCTGCCGTA -3′, reverse 5′-CGCAGTGAAGCCCATGGCAC-3′; MX1 forward: 5′-TTCCAGTCCAGCTCGGCAACA-3′, reverse 5′-TGGCTGGAGATGCGCTTGCTG-3′; RSAD2 forward: 5′-TCCTTTGTGCTGCCCCTTGAGGA-3′, reverse 5′-GCCCAGGTATTCTCCCCGGT-3′; CD169 forward: 5′-GCGATGCTGGCGTCTACA-3′, reverse 5′-ATTGGGTGTGTTGCAGACTAGTGT-3′; LGALS3BP forward: 5′-AATGAAACCAGGAGCACCCAC-3′, reverse 5′- CCTGCACATTCACGCTGATG-3′. Total RNA (200 ng) was reverse transcribed to cDNA using oligo(dT)_20_ primers and SuperScript III First Strand Synthesis Kit (Life Tech). SYBR green PCR reactions were performed on an ABI Viia7 instrument with the following cycle: 50C 2 min, 95C 10min; 95C 15 sec, 61C 1 min for 40 cycles; 95C 15 sec, 61C 1 min, 95C 15 sec for disassociation curves. The collected data were processed using EXCEL and a ΔΔCt method with a GAPDH reference. Gene expression relative to GAPDH was transformed to log_2_ to obtain normal distribution. Transformed data were used for correlation analysis.

### Flow Cytometry

T cell phenotypes were analyzed by triple staining PBMCs with fluorochrome-conjugated monoclonal antibodies for flow cytometric analyses. Fluorescein isothiocyanate (FITC) anti-CD4, FITC anti-CD8, Phycoerythrin (PE) anti-CD38 and Peridinin-cholorphyll protein (PerCP) anti- HLADR were obtained from BD. Isotype controls were fluorochrome-matched antibodies used to set cell gating and background staining. Whole blood was collected in sodium citrate tubes by venipuncture and PBMCs were enriched by Ficoll. PBMCs were stained with either FITC-anti-CD4, PE-anti-CD38, PerCP-anti-HLADR or FITC-anti-CD8, PE-anti-CD38, PerCP-anti-HLADR at 4C for 30 min in 2% mouse serum. Cells were then washed and fixed in 4% paraformaldehyde. Viable lymphocytes were gated based on forward and side scatter and T cell subsets CD4+ and CD8+ were analyzed for CD38 and HLADR. A minimum of 10,000 events were collected on a FACSCaliber (BD) and the frequency of CD38+ or HLADR+ T cells was determined by CellQuest software (BD).

### Assay for Plasma Lipopolysaccharide

Plasma endotoxin levels were quantified using the Pyrogene Recombinant Factor C Endotoxin Detection System (Lonza) according to manufacturer’s protocol. Plasma samples were isolated and stored in pyrogen-free tubes and dispensed and assayed in pyrogen-free labware.

### Statistical Analysis

Determination of differential gene expression and multiple testing correction/false discovery rate adjustments [17], [23] were performed using GeneSpring GX 7.3 software package (Agilent). Gene expression intensities were log_2_ transformed. Mean fold changes and standard errors for the mean fold changes were estimated using the log-transformed data. Results with continuous outcomes were analyzed using T tests and categorical outcomes were analyzed with chi-square methods and Fisher exact tests. Correlations between GDS and monocyte cDNA microarray gene expression data or T cell activation markers determined by flow cytometry were tested using Spearman’s rank correlation analysis. Pearson correlation coefficient analysis was used for comparison of qPCR and GDS. Means of groups for CD4 and CD8 data were compared with Student’s t test with multiple testing corrections [23]. Data were analyzed using statistical computing software developed by the R Foundation v2.12 [21].

## Results

### Demographic and Clinical Characteristics

This study uses two different cohorts in order to contrast monocyte gene expression in HIV monoinfected, HCV monoinfected and HIV/HCV coinfected individuals (Table 1). The HCV cohort was comprised of HCV monoinfected subjects (n = 19), HIV-suppressed coinfected subjects (n = 17) and healthy controls (n = 17). HIV-suppressed coinfected subjects were compliant on ART with undetectable HIV viral load (<50 copies/ml) for a six-month period prior to enrollment. For comparison, an HIV cohort was included consisting of subjects with an undetectable viral load (HIV_UD_; n = 14), high viral load (HIV; n = 22) and HIV seronegative healthy controls (n = 11). All subjects were male with no significant difference in either age or race. The CD4 T cell counts (mean±SD) in the HIV-suppressed coinfected and HIV_UD_ were similar at 501 (±243)×10^6^/L and 515 (±261)×10^6^/L, respectively.

**Table 1:** Subject clinical and demographic information.

	HCV cohort			HIV cohort		
	Controls	HCV	HIV/HCV	Controls	HIV a	HIV_UD_ b
N	17	19	17	11	22	14
Age (yr) c	53.4 (7.1)	56.6 (4.5)	54.5 (5.2)	53.0 (4.1)	49.9 (7.7)	51.6 (7.2)
Ethnicity d						
Asian	1	0	0	1	1	0
Black	4	6	6	1	6	2
Caucasian	11	11	10	8	12	11
Hispanic	1	2	1	1	3	1
HCV RNA (log_10_ IU/mL)^e^	NA	5.9 (0.8)	6.2 (0.5)	NA	NA	NA
HIV RNA (log_10_/mL)	NA	NA	undetectable	NA	5.0 (0.5)	undetectable
CD4 Count f	NA	NA	501 (243)	1038 (314)	215 (234)	515 (261)

Mean (± SD) except for ethnicity which is count.

a HIV_HVL_ ≥10,000 copies/ml.

b HIV_UD_ = undetectable viral load (<50 copies/ml).

c HIV is younger than HCV (anova p = 0.026, Tukey posthoc p = 0.011).

d *X*_2_ = 11.9, p = 0.919.

e Student t test p = 0.103.

f Anova p<0.001, HIV/HCV, HIV and HIV_UD_ were lower than HIV-Controls (Tukey posthoc p<0.001). HIV was lower than HIV/HCV and HIV_UD_ (Tukey posthoc p<0.05).

NA: not applicable.

Analyses of T cell activation markers CD38 and HLADR were performed on PBMCs isolated from the HCV cohort subjects including controls, HCV and HIV-suppressed coinfected. HIV monoinfected subjects with undetectable viral loads (HIV_UD_; n = 7) were recruited specifically for T cell analysis.

### Monocyte Gene Expression Profiles in HIV-suppressed Coinfected Subjects

Circulating CD14 monocytes constitute a heterogeneous cell population that are activated by viral and bacterial pathogens. Monocytes are also reactive to secondary factors such as cytokines elaborated from pathogen-activated cells making them ideal for assessing innate immune responses to HCV and HIV viral infections. For gene expression analysis, monocytes were rapidly isolated (<1 h) from PBMCs by CD14 positive selection at high purity to preserve fidelity of the *in vivo* gene expression profile. By analyzing exclusively monocytes, instead of PBMCs, we eliminated gene expression variability caused by individual differences in the ratio of leukocyte and lymphocyte populations. Data from the microarray analysis of the HCV cohort has been deposited in the NCBI GEO database (accession number GSE38542).

After collecting the monocyte gene expression data, we examined gene expression profiles in subjects having either HCV or HIV monoinfection and identified genes with the highest fold change compared to healthy controls. For HCV-monoinfected subjects, we identified 15 differentially expressed (DE) genes, which ranged from 24 to 3.7-fold change (Table 2). Genes with the highest DE included AREG, IL1A, ATF3 and CD83, which are listed showing cDNA microarray probe intensities along with associated Gene Ontology information. Similar analysis identified 15 DE genes in monocytes isolated from HIV_UD_-infected subjects (Table 2). In contrast to HCV-induced genes, none exceeded a 2-fold increase indicating that monocyte gene expression in HIV-infected subjects with suppressed viral replication is not activated and comparable to healthy controls. For comparison, we show that HIV viremia triggers substantial monocyte gene induction with the fold changes ranging from 116–5.4 in the top 15 DE genes (Table 2). Genes elevated in the HIV group such as IFI27, SIGLEC1, ETV7, RSAD2, IFI44L and LGALS3BP are all monocyte type 1 IFN response genes that have been identified in IFNα-treated monocytes and HIV-infected subjects with high viral loads [24]. If coinfection is a strictly additive response between HCV viremia and HIV_UD_, we would expect a monocyte profile in HIV-suppressed coinfected subjects that was the combination of the two viral responses.

**Table 2:** CD14 monocyte genes induced by viral infection.

*HCV*					
Symbol	GenBank	Control	HCV	FC^a^	GO Categories^b^
AREG	NM_001657	22 (109)	526 (5)	24.4	cell-cell signaling
NCOA7	W27126	9 (31)	126 (2)	13.5	regulation of transcription, DNA-dependent
IL1A	NM_000575	11 (11)	92 (5)	8.8	inflammatory response; anti-apoptosis
ANKDD1B	N81013	17 (38)	141 (1)	8.5	signal transduction
EIF1AX	N73881	13 (34)	115 (2)	8.5	translation; gene expression
CD83	NM_004233	76 (4)	544 (4)	7.1	immune response; signal transduction
IRF1	AB209624	78 (25)	553 (2)	7.1	transcription, DNA-dependent; I-kB kinase/NF-kB cascade
EGR3	NM_004430	62 (20)	356 (3)	5.8	cell-cell signaling; cell migration involved in sprouting angiogenesis
ME2	NM_002396	180 (20)	921 (2)	5.1	oxidation-reduction process
ATF3	NM_004024	433 (3)	2009 (3)	4.6	gluconeogenesis; regulation of transcription, DNA-dependent
PROS1	NM_000313	48 (15)	204 (2)	4.3	proteolysis
GPR183	NM_004951	1052 (5)	4337 (4)	4.1	immune response; G-protein coupled receptor signaling pathway
RASGEF1B	NM_152545	31 (13)	127 (3)	4.1	signal transduction
APOL2	NM_030882	33 (13)	129 (2)	3.9	lipid metabolic process; lipid transport
PNPLA2	X56789	140 (4)	521 (2)	3.7	lipid metabolic process; lipid catabolic process
***HIV_UD_***					
**Symbol**	**GenBank**	**Control**	**HIV_UD_**	**FC**	**GO Categories**
TET2	AK027819	1333 (3)	2690 (1)	2	myeloid cell differentiation
CCL5	NM_002985	420 (2)	831 (2)	2	chemotaxis; chronic inflammatory response
GZMA	NM_006144	192 (2)	373 (2)	1.9	immune response; apoptotic process; proteolysis
IL32	NM_004221	259 (2)	499 (1)	1.9	immune response; cell adhesion
CST7	NM_003650	111 (2)	213 (2)	1.9	immune response
GZMK	NM_002104	65 (2)	122 (1)	1.9	proteolysis
LGALS3BP	NM_005567	602 (2)	1082 (1)	1.8	defense response; cell adhesion
MORF4L2	NM_012286	282 (2)	503 (2)	1.8	regulation of cell growth; DNA repair
KLRD1	NM_002262	71 (2)	126 (2)	1.8	regulation of immune response
PRF1	NM_005041	60 (2)	106 (2)	1.8	immune response to tumor cell; apoptotic process
C2	NM_000063	371 (2)	613 (1)	1.7	innate immune response; proteolysis; complement activation
OTP	AI300650	171 (2)	279 (2)	1.6	positive regulation of neuroblast proliferation; nervous system development
KLF5	CB306177	197 (2)	318 (2)	1.6	angiogenesis; regulation of transcription, DNA-dependent
CRIPAK	NM_175918	270 (2)	430 (2)	1.6	negative regulation of protein kinase activity; ER-nucleus signaling pathway
MYC	NM_002467	562 (2)	892 (2)	1.6	MAPK cascade; B cell apoptosis; release of cytochrome c from mitochondria
***HIV***					
**Symbol**	**GenBank**	**Control**	**HIV**	**FC**	**GO Categories**
IFI27	T47364	26 (2)	2974 (4)	116	type I interferon-mediated signaling pathway; induction of apoptosis by extracellular signals
SIGLEC1	NM_023068	26 (2)	430 (2)	16.6	inflammatory response; cell adhesion; endocytosis
DEFB1	NM_005218	8 (21)	123 (3)	14.7	chemotaxis; immune response
ETV7	NM_016135	28 (9)	383 (2)	13.8	regulation of transcription
SERPING1	NM_000062	237 (2)	2006 (2)	8.5	inflammatory/immune response; complement activation
GBP1P1	CA309689	134 (2)	984 (2)	7.3	
IFIT1	NM_001548	646 (1)	4714 (2)	7.3	DNA replication, recombination and repair
RSAD2	NM_080657	201 (1)	1408 (2)	7	immune response; defense response to virus
LGALS3BP	NM_005567	602 (2)	4181 (2)	6.9	defense response; cell adhesion
CXCL11	NM_005409	54 (2)	349 (2)	6.4	chemotaxis; inflammatory/immune response
IFI44L	NM_006820	1169 (1)	7396 (2)	6.3	immune response
KLHDC7B	AA854620	387 (1)	2334 (2)	6	protein binding
CFB	NM_001710	33 (3)	196 (2)	5.9	innate immune response; complement activation; proteolysis
ATP10A	BC052251	24 (3)	139 (2)	5.8	phospholipid transport; regulation of cell shape
CCL8	NM_005623	57 (2)	307 (2)	5.4	monocyte chemotaxis; angiogenesis

a Fold change based on mean probe intensity.

b Geng002e Ontology.

**Figure 1:**
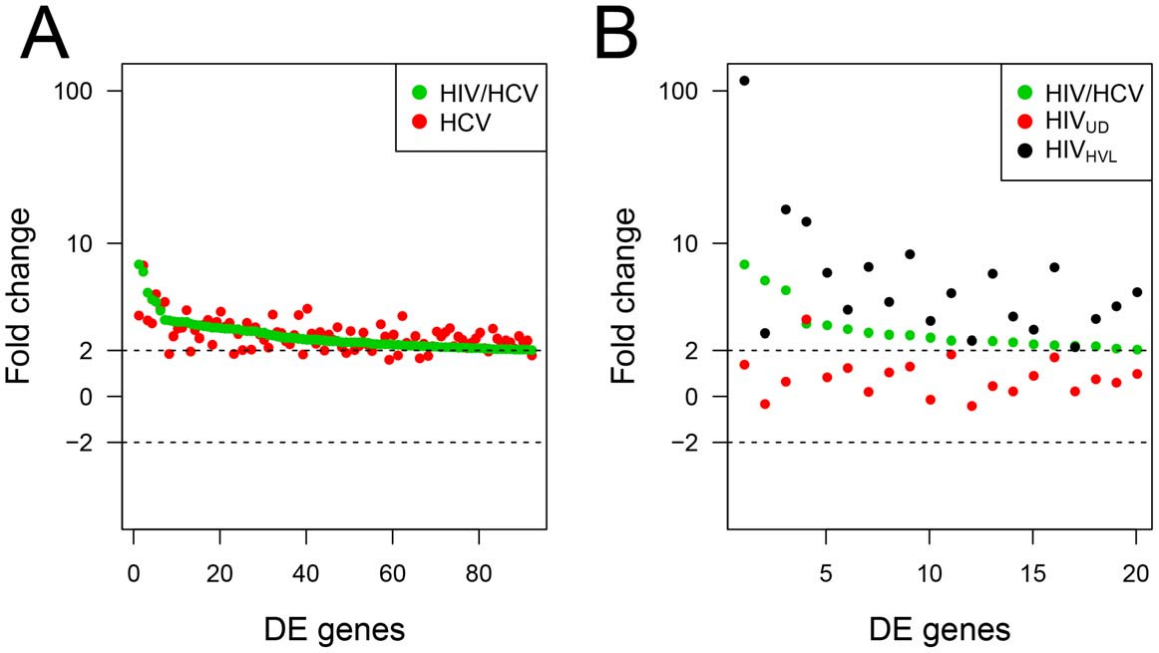
Monocyte gene expression in HIV/HCV coinfection compared to HCV and HIV monoinfection. CD14 monocytes from HIV-suppressed coinfected, HCV and HIV monoinfected subjcects were analyzed using cDNA high-density microarrays. Differentially expressed genes (DE; compared to healthy controls) were transformed and displayed as log fold change. Monocyte DE genes in the HIV-suppressed coinfected subjects (induced ≥2-fold and sorted in decreasing fold change) were matched with corresponding genes in monoinfected subjects. (**A**) 92 DE genes from HIV-suppressed coinfected subjects (green) are plotted based on fold change and matched with corresponding genes in HCV-infected subjects (red). (**B**) Fold change in 20 DE genes in HIV-suppressed coinfected subjects (green) are shown with corresponding genes in HIV-infected (black; HVL; ≥10,000 copies/ml) and HIV-infected subjects with undetectable viral loads (red; HIV_UD_; <50 copies/ml).

Next we determined how coinfection impacts monocyte expression compared to monoinfection by either HCV or HIV. For this, monocyte genes DE >2-fold in HIV-suppressed coinfected subjects were matched with corresponding genes in HCV monoinfected subjects (Fig. 1A). This analysis showed that 92 monocyte genes expressed in the HIV-suppressed coinfected subjects (green circles) had essentially the same level of expression in the HCV monoinfected subjects (red) indicating that coinfection does not markedly increase HCV-associated gene expression. Likewise, we performed analysis comparing gene expression in HIV-suppressed coinfected subjects (green) with DE genes from both HIV_UD_ (red) and HIV monoinfected subjects (black) (Fig. 1B). In this case, gene expression in the coinfection did not track with the HIV_UD_ expression profile, even though that would be expected with undetectable viral loads. Instead, gene expression was intermediate between the HIV undetected and high viral load profiles, suggesting that coinfection triggers a cellular activation profile with similarity to an HIV-elicited response.

**Figure 2:**
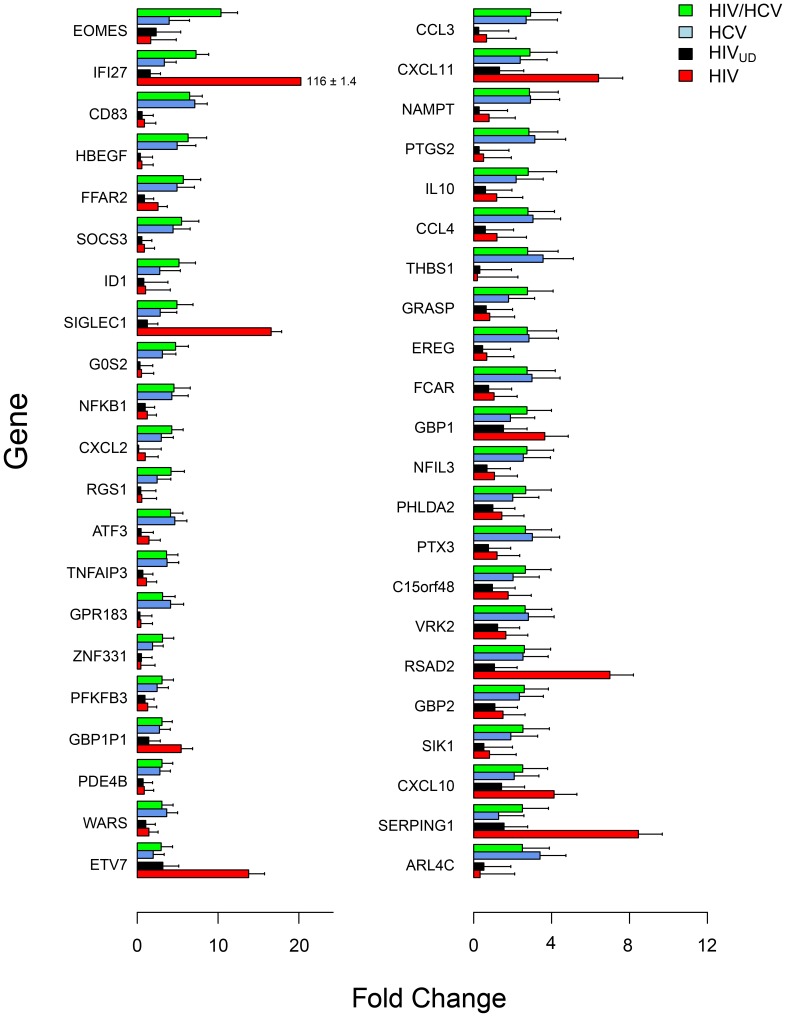
Comparative gene expression in CD14 monocytes by cDNA microarray analysis. Forty-three differentially expressed genes (>2.5-fold) in HIV-suppressed coinfected subjects compared to controls were sorted based on fold change (green; n = 17) and displayed with corresponding genes in subjects infected with HCV (blue, n = 19), HIV_UD_ (black; n = 14; <50 copies/ml) and HIV (red; n = 22; >10,000 copies/ml)**.** Gene expression intensities were log_2_ transformed and mean fold change and standard error was estimated based on log-transformed data.

For a more comprehensive analysis, cDNA microarray expression data were sorted based on monocyte gene expression in HIV-suppressed coinfected subjects. We identified 43 DE genes >2.5-fold with in HIV-suppressed coinfected subjects (green) relative to controls (Fig. 2). For each gene from the HIV-suppressed coinfected subjects, the corresponding gene expression was determined in HCV (blue), HIV_UD_ (black) and HIV-infected subjects (red) (Fig. 2; Table S1). Based on comparative expression, monocyte genes in HIV-suppressed coinfected subjects showed profiles similar to HCV, HIV or an intermediate response. Genes such as AREG, IL1A, ATF3 and CD83, which were elevated in the HCV monoinfected subjects but not in the HIV subjects, indicated an HCV-specific response. Then there were the HIV-related genes, IFI27, SIGLEC1, SERPING1 and LGALS3BP, which were elevated in HIV-suppressed coinfected and HIV subjects but were lower in HCV subjects. The HIV-associated genes were all type 1 IFN response genes that were induced in HIV-suppressed coinfected and HIV but not in HIV_UD_ suggesting that coinfection triggers a chronic activation state that in this case is not related to viral load.

### Cognitive Impairment Correlates with Monocyte Activation but not Plasma LPS

To determine if coinfection was associated with an elevated risk of cognitive impairment, subjects in the study received a battery of neuropsychological tests which are widely used to detect impairment in HIV-infected individuals including diminished cognitive skills that are critical for daily living [19], [25]. Neuropsychological tests assessing 7 domains were summarized in the GDS (Table 3), which integrates and normalizes relevant NP test results into a unitary global score [26], [27]. This method effectively discriminates between normal and neurocognitively impaired individuals in both control and HIV-infected individuals according to clinical rating standards and compensates for limitations of group mean comparisons by detecting subtle cognitive impairment affecting a minority of individuals [27]. It assumes equal weighting of standard scores while giving relatively less weight to performances within and above normal limits. Individual GDS were computed by correcting NP standard scores (T scores) for educational level, gender, and age. Averaged deficit scores generated the GDS [27]. Using GDS, mild impairment is defined as ≥0.5–1.5. In this cohort, GDS in control subjects averaged 0.30 (0.31), HCV 0.46 (0.34), HIV_UD_ 0.45 (0.36) and HIV-suppressed coinfected 0.76 (0.47) (Table 3) [18]. HIV-suppressed coinfected subjects had significantly higher GDS compared to controls (*p* = 0.02), HCV monoinfected (*p*<0.05) or HIV_UD_ (*p*<0.05) suggesting that cognitive deficits were associated with coinfection. This finding was consistent with a recent report of increased cognitive impairment in coinfected subjects showing a higher prevalence of neurologic disease [10].

**Table 3:** Subject T scores for neuropsychological domains and combined global deficit scores.

Domain	C^a^	HCV	HIV/HCV^b^	HIV_UD_ c
Attention/Working Memory	57.1 (17.5)	45.4 (7.6)	46.6 (6.6)	44.5 (9.9)
Information Processing Speed	47.6 (8.8)	44.6 (6.6)	44.0 (7.3)	47.0 (7.7)
Executive Function	46.1 (6.9)	44.9 (5.9)	41.4 (5.6)	44.6 (8.1)
Fine Motor Function	50.6 (12.7)	47.6 (7.6)	42.1 (5.3)	57.0 (10.9)
Verbal Fluency	47.4 (9.2)	47.6 (8.1)	46.1 (8.6)	49.9 (8.4)
Visual Learning/Memory	45.5 (12.8)	39.7 (12.7)	30.9 (10.0)	45.9 (12.6)
Verbal Learning/Memory	53.6 (10.3)	47.2 (8.2)	41.5 (8.2)	53.2 (7.1)
**Global deficit score (GDS)**	**0.30** **(0.31)**	**0.46** **(0.34)**	**0.76** **(0.47)**	**0.45** **(0.36)**

Mean (SD).

a Healthy controls.

b HIV-suppressed (<50 copies/ml) coinfected.

c HIV undetected (<50 copies/ml).

**Figure 3:**
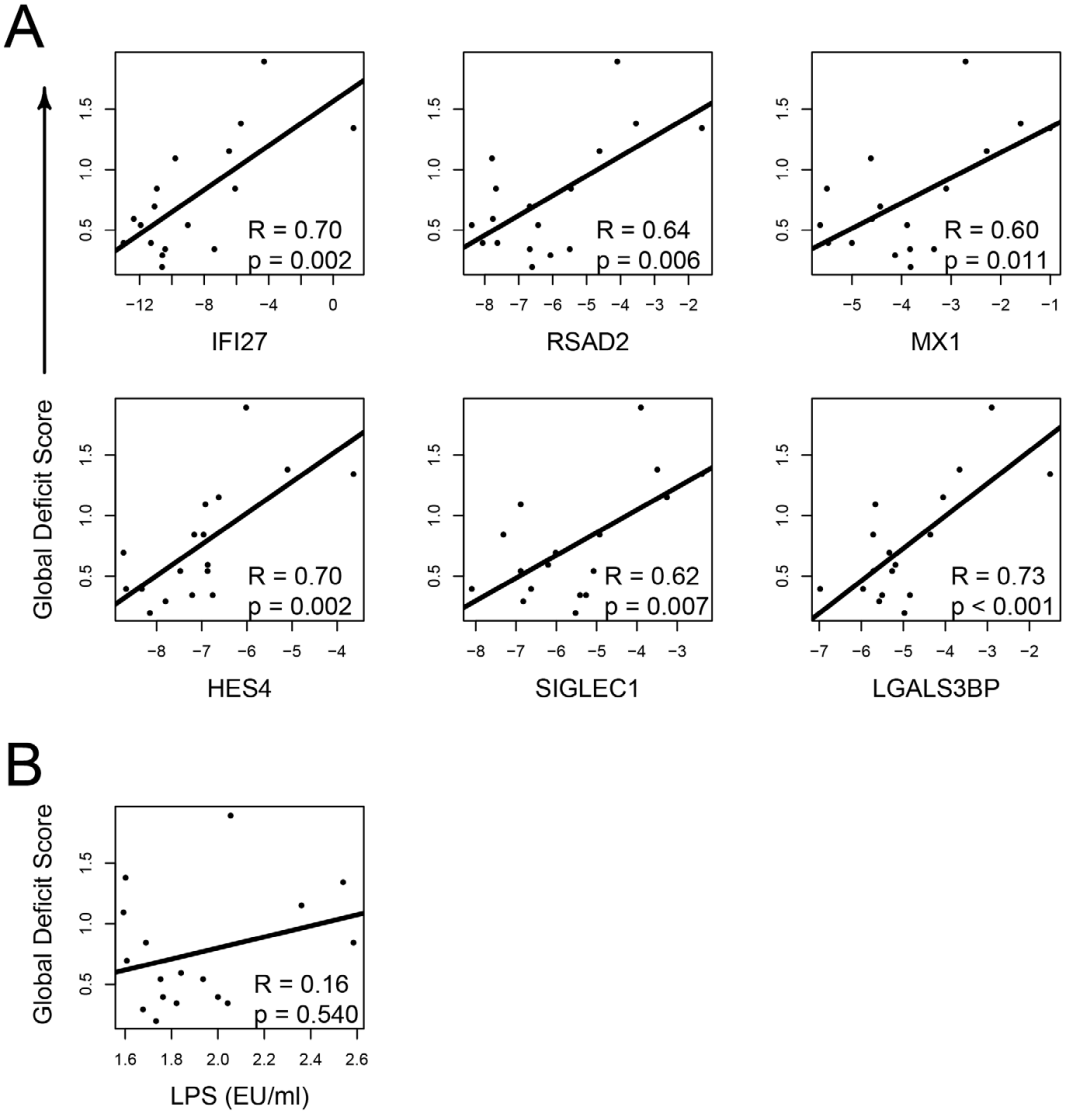
Upregulation of HIV-related monocyte genes correlated with GDS in HIV-suppressed coinfected subjects. Global deficit scores were calculated from neuropsychological tests assessing seven domains where GDS >0.5 designates cognitive impairment. (**A**) Expression of six genes identified by cDNA microarrays were validated by qPCR and reanalyzed for correlated expression with GDS. Gene expression (x axis) was determined relative to GAPDH and transformed to log_2_. All genes tested correlated with GDS (Pearson coefficient analysis). (**B**) Plasma endotoxin (EU/ml) in HIV-suppressed coinfected subjects showed no correlation with GDS (Spearman rank correlation).

To investigate whether monocyte activation observed in HIV-suppressed coinfected subjects was related to worsening neurocognitive status, 43 DE monocyte genes (Fig. 2) upregulated >2-fold in HIV-suppressed coinfected subjects were tested for correlations with subject’s GDS. Spearman rank coefficient analysis identified eight genes, IF127, RSAD2, MX1 ETV7, SIGLEC1, LGALS3BP, C1QC and HES4 that significantly correlated with GDS (*p*<0.05). Six of the genes identified as significant from the microarray data were then validated by qPCR and again correlated with the GDS (Pearson coefficient) (Fig. 3A). All six genes were found to be significant and five out of six were type 1 IFN response genes with the exception of HES4.

Lipopolysaccharide has been linked to HIV disease progression [28] and associated with cognitive impairment [29]. To explore a potential link between inflammation and cognitive impairment in HIV-suppressed coinfected subjects, we first determined plasma endotoxin concentrations (endotoxin units, EU; ±SD) and found both HIV-suppressed coinfected (1.9±0.3 EU/ml) and HIV_UD_ (2.7±1.2 EU/ml) subjects had significantly elevated levels compared to controls (1.7±0.3 EU/ml) (p<0.05), while HCV monoinfected subjects (1.8±0.3 EU/ml) were no different than controls. There was no correlation when plasma endotoxin levels were compared with GDS in the HIV-suppressed coinfected group indicating that LPS was not responsible for lower cognition (Fig. 3B).

### T cell Activation in HIV/HCV Infection

**Figure 4:**
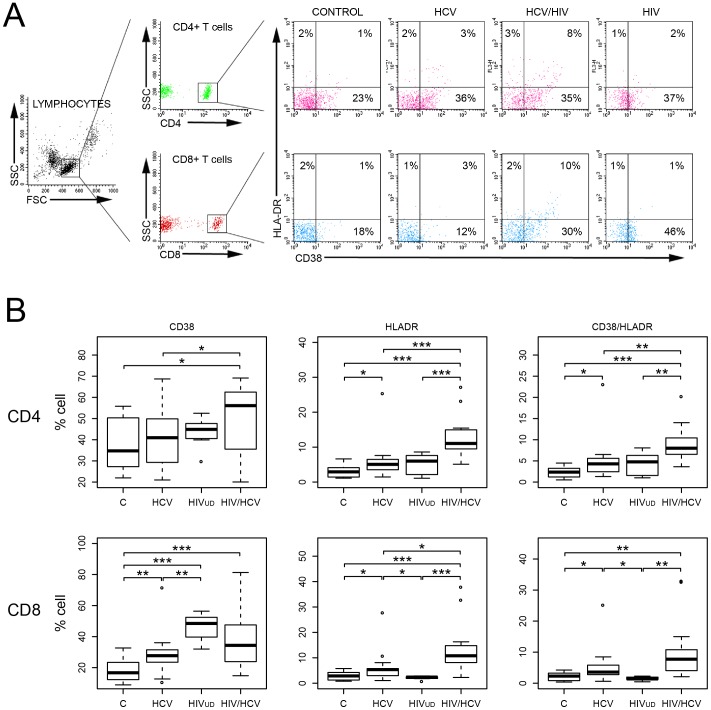
Flow cytometric analysis of activation markers on CD4 and CD8 T cells. (**A**) Representative analysis of PBMCs from infected and control subjects. Lymphocytes were gated on forward and side scatter and T cells were subsequently gated on CD4 or CD8 expression. Quadrants were set using isotype controls for CD4, CD8, CD38 and HLADR. Data represent triple-stained cells with a minimum of 10,000 counts and shown as percent of cells expressing CD4/CD38/HLADR and CD8/CD38/HLADR above the isotype control. T cells were gated as CD4 or CD8 and subsequently analyzed for CD38 and HLADR. Coinfection (HCV/HIV) triggered increased HLADR and CD38/HLADR staining. (**B**) Quantitative analysis of both T cell subsets for CD38 and HLADR and double-stained for CD38/HLADR. For CD4 and CD8 T cells, subjects were controls (C), HCV, HIV_UD_ and HIV-suppressed coinfected (HIV/HCV). Significance determined by Student’s t-test (*p<0.05, **p<0.01, ***p<0.001).

Immune activation in subjects chronically infected with HIV is associated with elevated T cell activation markers. We determined the extent of T cell activation in HIV-suppressed coinfected, HCV, HIV_UD_ and controls by analyzing CD38 and HLADR expression on both CD4 and CD8 T cells. Freshly isolated PBMCs were analyzed by flow cytometry and lymphocytes were gated based on forward and side scatter (Fig. 4A). Representative flow cytometry data illustrates the distribution of cells triple stained for CD4, CD38 and HLADR and for CD8, CD38 and HLADR. Collectively, the percent of positive cells in the CD4 and CD8 subsets are shown as separately staining CD38 and HLADR cells, and as co-expressing CD38/HLADR cells in healthy controls (n = 17) and subjects with HCV (n = 17), HIV_UD_ (n = 8) or HIV-suppressed coinfected (n = 17) (Fig. 4B). CD38 expression in CD4 cells was elevated in HIV-suppressed coinfected subjects compared to HCV monoinfection and controls (*p*<0.05) but not significantly above HIV_UD_. For HLADR, significantly higher expression was observed in HIV-suppressed coinfected compared to the other groups (*p*<0.001), which was also true for CD38/HLADR expressing cells (*p*<0.01). In the CD8 subset, HIV-suppressed coinfected subjects showed higher CD38 expression compared to controls (*p*<0.001) but was not significantly elevated in either HCV or HIV_UD_ groups. For HLADR and CD38/HLADR, results were similar yet not as dramatic as the CD4 subset, with coinfection associated with significantly higher expression. Data show that HIV_UD_ and coinfection in both T cell subsets have overlapping CD38 expression profiles, which might be caused by HIV-related chronic immune activation even in subjects with controlled viral load. In contrast, HLADR is a better gauge of coinfection-related activation regardless of the T cell subset. To determine whether T cell activation might be linked to cognition in HIV-suppressed coinfected subjects, we tested for possible correlations between CD38 or HLADR and GDS. Spearman rank coefficient analysis did not reveal any interconnection (data not shown). So, while T cell activation points to a perturbation in the T cell compartment related to coinfection, T cell activation appears to be independent of cognitive dysfunction.

## Discussion

In this study we examined both monocyte and T cell populations to understand the impact of HIV/HCV coinfection on peripheral immune cells and how that may relate to cognition. We selected CD14 monocytes for gene expression analysis based on their immune response characteristics, including sensitivity to LPS, and because they could be isolated rapidly at high purity from whole blood thereby preserving their *in vivo* phenotype. For these analyses, we included monocyte gene expression data obtained from HIV-infected individuals with viremia or undetectable viral loads that was previously published [17]. Gene expression analysis of monocytes from HCV and HIV_UD_ subjects revealed distinct expression profiles with significant induction of monocyte genes in HCV-infected subjects. In contrast, monocyte expression profiles in HIV_UD_ subjects were similar to healthy controls. Furthermore, monocyte genes upregulated in HCV subjects were comparably expressed in HIV-suppressed coinfected subjects, while HIV-related genes in the HIV-suppressed coinfected were similar to those in HIV subjects but not induced in HIV_UD_ subjects. A possible explanation for the activated profile in HIV-suppressed coinfected subjects is that HIV primes monocytes to be hypersensitive to agonists that activate the innate immune response [30]. While HCV in chronically infected subjects did not elicit an HIV expression profile, HCV in the context of HIV priming may trigger monocyte activation. Monocytes harvested from HIV-infected individuals and analyzed for gene expression by us and others have identified a type 1 IFN response where the level of activation was dependent on viral load [17], [31], [32], [33]. However, HIV viral load is unlikely driving the IFN response in HIV-suppressed coinfected subjects since HIV was undetectable. Our finding is consistent with an earlier report of an aberrant type1 IFN response in HIV-suppressed coinfected individuals also identifying upregulation of IFI27, IFI44 and MX2 in PBMCs [15], [34]. Interestingly, immune activation was reduced following effective peg-IFN/ribavirin treatment in individuals who achieved HCV sustained viral response [14]. These results are consistent with monocyte activation being triggered by factors other than HIV viral load and implicate HCV infection through, as yet, an undefined mechanism triggering immune activation in HIV-suppressed coinfected subjects.

In this cohort, we found that coinfection was associated with significantly greater cognitive dysfunction, greater than that in either HCV or HIV monoinfected subjects. Numerous studies have evaluated the neuropsychological status of HIV-suppressed coinfected individuals and the literature is divided between studies which did not find a difference between HIV-suppressed coinfected and HCV monoinfected patients [35], [36], [37] and those which found worse cognitive performance in the HIV-suppressed coinfected [9], [10], [38]. Even when impairment was identified, it is often judged to be mild, which implies that subtle differences in cohort recruitment as well as the choice of particular neuropsychological tests may have a considerable impact on the cognitive characterization of HIV-suppressed coinfected subjects. In this cross-sectional study, we attempted to minimize confounding factors by recruiting HIV-suppressed coinfected subjects with undetectable HIV viral loads who were receiving stable medical care, had no significant liver disease and were infected with HCV genotype 1 with no current drug or alcohol abuse [18]. Subjects were assessed for neuropsychological deficits in seven domains thereby increasing sensitivity with resulting T scores converted into GDS. Using Spearman rank coefficient analysis, we identified eight genes, IF127, RSAD2, MX1, ETV7, SIGLEC1, LGALS3BP, C1QC and HES4 from HIV-suppressed coinfected subjects that correlated with GDS thereby linking an expression profile with poorer cognition. Seven were type 1 IFN-related genes while HES4 was upregulated in both HCV and HIV monoinfection and all of the genes were expressed at levels above that found in the HIV_UD_. The Hairy/Enhancer of Split 1 (HES1) gene product is a transcriptional repressor implicated in the control of proliferating neural stem cells [39] which itself is regulated by notch signaling [40]. While HES4 was not induced in monocytes treated with IFNα [17], it was upregulated 7-fold in PBMCs treated with immune complexes that cause inflammation in systemic lupus erythematosus [41]. Four of the genes, IFI27, ETV7, SIGLEC1 and LGALS3BP were previously identified in activated monocytes isolated from HIV subjects with viremia where their expression correlated with lower brain metabolite N-acetylaspartate levels in frontal white matter as determined by ^1^H magnetic resonance spectroscopy [42]. Together these observations point to a relationship connecting coinfection and monocyte activation that impacts cognitive function perhaps by altering brain metabolite levels.

Lipopolysaccharide is a potent proinflammatory agent possibly linked to cognitive impairment. During HIV infection, the gastrointestinal barrier is compromised due to selective loss of CD4 T cells with subsequent bacterial translocation and elevated endotoxin levels in the blood [28]. Plasma LPS is elevated in HIV-infected subjects compared to healthy controls [43] and was found to correlate with HIV-associated dementia independent of viral load [29]. In coinfection, impaired LPS detoxification due to liver damage could contribute to high immune activation levels, which is supported by evidence that microbial translocation is associated with cirrhosis [44]. We examined plasma LPS levels in our cohort and found significantly higher LPS in the HIV-suppressed coinfected population compared to healthy controls. We also tested for a correlation between plasma LPS concentration and GDS in the HIV-suppressed coinfected and found no relationship. Based on these observations, LPS does not appear to be a factor causing cognitive impairment in HIV-suppressed coinfected individuals.

We examined the T cell compartment to determine if coinfection was related to an activated phenotype. HIV drives persistent immune activation typified by a higher percentage of T cells expressing activation markers [45]. The most effective correlate of HIV disease progression is upregulation of CD38 on CD8 T cells [46] with one of the consequences being activation-induced apoptosis in CD4 and CD8 T cells [47]. While effective ART in HIV-infected subjects dramatically lowers activation by suppressing HIV viral replication and allowing partial reconstitution of immune function, low-level persistent activation remains [48], [49]. Incomplete restoration of normal CD38 expression in HIV_UD_ subjects was evident in our subjects as well, particularly in the CD8 cells, which expressed significantly elevated CD38 compared to controls and HCV monoinfected subjects. In our cohort, HLADR was the most effective marker for differentiating coinfection in both CD4 and CD8 subsets demonstrating significantly higher expression than either HCV or HIV monoinfection, which extended to the CD38/HLADR population as well. Others who have investigated these markers in HIV-suppressed coinfected subjects have likewise identified activation in the T cell compartment but with somewhat different results. Kovacs et al. found elevated CD38/HLADR in CD8 but not in the CD4 subset [13] while Gonzalez et al., examined only CD38 expression which was increased in both CD4 and CD8 subsets in HIV-suppressed coinfected compared to HCV and HIV monoinfection [14]. It is important to consider this activation in context of controlled HIV viral load, which in our cohort was undetectable. This suggests an alternative mechanism for peripheral activation in coinfection since viral suppression of HIV is normally sufficient to lower T cell activation markers. In addition, T cell activation did not correlate with cognitive impairment in HIV-suppressed coinfected subjects, which was contrary to expectation. This suggests that monocyte activation in coinfection is not intrinsically associated with T cell activation.

Irrefutable evidence that coinfection increases the risk of cognitive impairment remains elusive. Published reports that detect impairment are counterbalanced by studies that fail to find significant association with coinfection. Considered separately, there is no question of HIV’s role in neurodegeneration and cognition impairment particularly when viral loads are not suppressed. With effective ART, neuropathogenesis has been reduced and cognition improved and yet HIV-associated neurocognitive disorders remain [50]. Findings of diminished cognition are equivocal regarding HCV, while more compelling in coinfection. We are the first to report a monocyte activation profile that correlates with cognitive impairment in the HIV-suppressed coinfected population. This implies a risk of impairment even when HIV viral loads are effectively suppressed. Recent improvements in HCV therapy include the addition of highly effective HCV protease inhibitors to the standard peg-IFN/ribavirin therapy [51]. This is expected to increase the number of coinfected individuals who are treated and ultimately the number who achieve a sustained viral response (SVR). By effectively suppressing HCV, we anticipate that monocyte activation will decrease with subsequent cognitive improvement in those who previously exhibited a type 1 monocyte expression profile. This conjecture needs to be tested using a longitudinal treatment study that could assess immune activation and cognitive impairment in individuals who maintain SVR.

## Supporting Information

Table S1 cDNA microarray probe intensities for monocyte genes induced in coinfected subjects (>2 fold change).
(DOCX)

## References

[1] Alter MJ (2006) Epidemiology of viral hepatitis and HIV co-infection. J Hepatol 44: S6–9. 16352363 10.1016/j.jhep.2005.11.004

[2] Anderson KB, Guest JL, Rimland D (2004) Hepatitis C virus coinfection increases mortality in HIV-infected patients in the highly active antiretroviral therapy era: data from the HIV Atlanta VA Cohort Study. Clin Infect Dis 39: 1507–1513. 15546088 10.1086/425360

[3] Koziel MJ, Peters MG (2007) Viral hepatitis in HIV infection. N Engl J Med 356: 1445–1454. 17409326 10.1056/NEJMra065142 PMC4144044

[4] Operskalski EA, Kovacs A (2011) HIV/HCV co-infection: pathogenesis, clinical complications, treatment, and new therapeutic technologies. Curr HIV/AIDS Rep 8: 12–22. 21221855 10.1007/s11904-010-0071-3 PMC3035774

[5] Schouten J, Cinque P, Gisslen M, Reiss P, Portegies P (2011) HIV-1 infection and cognitive impairment in the cART era: a review. AIDS 25: 561–575. 21160410 10.1097/QAD.0b013e3283437f9a

[6] McAndrews MP, Farcnik K, Carlen P, Damyanovich A, Mrkonjic M, et al (2005) Prevalence and significance of neurocognitive dysfunction in hepatitis C in the absence of correlated risk factors. Hepatology 41: 801–808. 15793853 10.1002/hep.20635

[7] Weissenborn K, Krause J, Bokemeyer M, Hecker H, Schuler A, et al (2004) Hepatitis C virus infection affects the brain-evidence from psychometric studies and magnetic resonance spectroscopy. J Hepatol 41: 845–851. 15519659 10.1016/j.jhep.2004.07.022

[8] Aronow HA, Weston AJ, Pezeshki BB, Lazarus TS (2008) Effects of coinfection with HIV and hepatitis C virus on the nervous system. AIDS Read 18: 43–48. 18240452

[9] Hinkin CH, Castellon SA, Levine AJ, Barclay TR, Singer EJ (2008) Neurocognition in individuals co-infected with HIV and hepatitis C. J Addict Dis. 27: 11–17. 10.1300/j069v27n02_02 PMC2886797 18681187

[10] Vivithanaporn P, Nelles K, DeBlock L, Newman SC, Gill MJ, et al (2012) Hepatitis C virus co-infection increases neurocognitive impairment severity and risk of death in treated HIV/AIDS. J Neurol Sci 312: 45–51. 21925684 10.1016/j.jns.2011.08.025

[11] Silvestri G, Paiardini M, Pandrea I, Lederman MM, Sodora DL (2007) Understanding the benign nature of SIV infection in natural hosts. J Clin Invest 117: 3148–3154. 17975656 10.1172/JCI33034 PMC2045617

[12] Hazenberg MD, Otto SA, van Benthem BH, Roos MT, Coutinho RA, et al (2003) Persistent immune activation in HIV-1 infection is associated with progression to AIDS. AIDS 17: 1881–1888. 12960820 10.1097/00002030-200309050-00006

[13] Kovacs A, Al-Harthi L, Christensen S, Mack W, Cohen M, et al (2008) CD8(+) T cell activation in women coinfected with human immunodeficiency virus type 1 and hepatitis C virus. J Infect Dis 197: 1402–1407. 18444798 10.1086/587696 PMC2443164

[14] Gonzalez VD, Falconer K, Blom KG, Reichard O, Morn B, et al (2009) High levels of chronic immune activation in the T-cell compartments of patients coinfected with hepatitis C virus and human immunodeficiency virus type 1 and on highly active antiretroviral therapy are reverted by alpha interferon and ribavirin treatment. J Virol 83: 11407–11411. 19710147 10.1128/JVI.01211-09 PMC2772767

[15] Kottilil S, Yan MY, Reitano KN, Zhang X, Lempicki R, et al (2009) Human immunodeficiency virus and hepatitis C infections induce distinct immunologic imprints in peripheral mononuclear cells. Hepatology 50: 34–45. 19551908 10.1002/hep.23055 PMC2736098

[16] Pulliam L, Sun B, Rempel H (2004) Invasive chronic inflammatory monocyte phenotype in subjects with high HIV-1 viral load. J Neuroimmunol 157: 93–98. 15579285 10.1016/j.jneuroim.2004.08.039

[17] Rempel H, Sun B, Calosing C, Pillai SK, Pulliam L (2010) Interferon-alpha drives monocyte gene expression in chronic unsuppressed HIV-1 infection. AIDS 24: 1415–1423. 20495440 10.1097/QAD.0b013e32833ac623 PMC2991092

[18] Sun B, Linda A, Rempel H, Monto A, Pulliam L (2012) Differential cognitive impairment in HCV coinfected men with controlled HIV compared to HCV monoinfection. J Acquir Immune Defic Syndr: In press. 10.1097/QAI.0b013e31827b61f1 PMC3587125 23187938

[19] Sun B, Abadjian L, Rempel H, Calosing C, Rothlind J, et al (2010) Peripheral biomarkers do not correlate with cognitive impairment in highly active antiretroviral therapy-treated subjects with human immunodeficiency virus type 1 infection. J Neurovirol 16: 115–124. 20307252 10.3109/13550280903559789

[20] Schroeder A, Mueller O, Stocker S, Salowsky R, Leiber M, et al (2006) The RIN: an RNA integrity number for assigning integrity values to RNA measurements. BMC Mol Biol 7: 3. 16448564 10.1186/1471-2199-7-3 PMC1413964

[21] Ihaka R, Gentleman R (1996) R: A Language for Data Analysis and Graphics. Journal of Computational and Graphical Statistics 5: 299–314.

[22] Gentleman RC, Carey VJ, Bates DM, Bolstad B, Dettling M, et al (2004) Bioconductor: open software development for computational biology and bioinformatics. Genome Biol 5: R80. 15461798 10.1186/gb-2004-5-10-r80 PMC545600

[23] Benjamini Y, Hochberg Y (1995) Controlling the false discovery rate: a practical and powerful approach to multiple testing. Journal of the Royal Statistical Society, Series B (Methodological) 57: 125–133.

[24] Rempel H, Calosing C, Sun B, Pulliam L (2008) Sialoadhesin expressed on IFN-induced monocytes binds HIV-1 and enhances infectivity. PLoS ONE 3: e1967. 18414664 10.1371/journal.pone.0001967 PMC2288672

[25] Heaton RK, Marcotte TD, Mindt MR, Sadek J, Moore DJ, et al (2004) The impact of HIV-associated neuropsychological impairment on everyday functioning. J Int Neuropsychol Soc 10: 317–331. 15147590 10.1017/S1355617704102130

[26] Carey CL, Woods SP, Gonzalez R, Conover E, Marcotte TD, et al (2004) Predictive validity of global deficit scores in detecting neuropsychological impairment in HIV infection. J Clin Exp Neuropsychol 26: 307–319. 15512922 10.1080/13803390490510031

[27] Heaton RK, Grant I, Butters N, White DA, Kirson D, et al (1995) The HNRC 500–neuropsychology of HIV infection at different disease stages. HIV Neurobehavioral Research Center. J Int Neuropsychol Soc 1: 231–251. 9375218 10.1017/s1355617700000230

[28] Brenchley JM, Price DA, Schacker TW, Asher TE, Silvestri G, et al (2006) Microbial translocation is a cause of systemic immune activation in chronic HIV infection. Nat Med 12: 1365–1371. 17115046 10.1038/nm1511

[29] Ancuta P, Kamat A, Kunstman KJ, Kim EY, Autissier P, et al (2008) Microbial translocation is associated with increased monocyte activation and dementia in AIDS patients. PLoS ONE 3: e2516. 18575590 10.1371/journal.pone.0002516 PMC2424175

[30] Brown JN, Kohler JJ, Coberley CR, Sleasman JW, Goodenow MM (2008) HIV-1 activates macrophages independent of Toll-like receptors. PLoS One 3: e3664. 19048100 10.1371/journal.pone.0003664 PMC2585009

[31] Tilton JC, Johnson AJ, Luskin MR, Manion MM, Yang J, et al (2006) Diminished production of monocyte proinflammatory cytokines during human immunodeficiency virus viremia is mediated by type I interferons. J Virol 80: 11486–11497. 17005663 10.1128/JVI.00324-06 PMC1642603

[32] Mandl JN, Barry AP, Vanderford TH, Kozyr N, Chavan R, et al (2008) Divergent TLR7 and TLR9 signaling and type I interferon production distinguish pathogenic and nonpathogenic AIDS virus infections. Nat Med 14: 1077–1087. 18806803 10.1038/nm.1871

[33] Stylianou E, Aukrust P, Bendtzen K, Muller F, Froland SS (2000) Interferons and interferon (IFN)-inducible protein 10 during highly active anti-retroviral therapy (HAART)-possible immunosuppressive role of IFN-alpha in HIV infection. Clin Exp Immunol 119: 479–485. 10691920 10.1046/j.1365-2249.2000.01144.x PMC1905596

[34] Lempicki RA, Polis MA, Yang J, McLaughlin M, Koratich C, et al (2006) Gene expression profiles in hepatitis C virus (HCV) and HIV coinfection: class prediction analyses before treatment predict the outcome of anti-HCV therapy among HIV-coinfected persons. J Infect Dis 193: 1172–1177. 16544259 10.1086/501365

[35] Clifford DB, Smurzynski M, Park LS, Yeh TM, Zhao Y, et al (2009) Effects of active HCV replication on neurologic status in HIV RNA virally suppressed patients. Neurology 73: 309–314. 19636051 10.1212/WNL.0b013e3181af7a10 PMC2715213

[36] Perry W, Carlson MD, Barakat F, Hilsabeck RC, Schiehser DM, et al (2005) Neuropsychological test performance in patients co-infected with hepatitis C virus and HIV. AIDS 19 Suppl 3S79–84. 16251832 10.1097/01.aids.0000192074.18691.31

[37] Crystal H, Kleyman I, Anastos K, Lazar J, Cohen M, et al (2012) Effects of hepatitis C and HIV on cognition in women: data from the Women's Interagency HIV Study. J Acquir Immune Defic Syndr 59: 149–154. 22107817 10.1097/QAI.0b013e318240566b PMC3319079

[38] Garvey LJ, Pavese N, Ramlackhansingh A, Thomson E, Allsop JM, et al (2012) Acute HCV/HIV Coinfection Is Associated with Cognitive Dysfunction and Cerebral Metabolite Disturbance, but Not Increased Microglial Cell Activation. PLoS One 7: e38980. 22808022 10.1371/journal.pone.0038980 PMC3395624

[39] El Yakoubi W, Borday C, Hamdache J, Parain K, Tran HT, et al.. (2012) Hes4 Controls Proliferative Properties of Neural Stem Cells During Retinal Ontogenesis. Stem Cells. 10.1002/stem.1231 PMC3549485 22969013

[40] Kageyama R, Ohtsuka T, Kobayashi T (2007) The Hes gene family: repressors and oscillators that orchestrate embryogenesis. Development 134: 1243–1251. 17329370 10.1242/dev.000786

[41] Santer DM, Wiedeman AE, Teal TH, Ghosh P, Elkon KB (2012) Plasmacytoid dendritic cells and C1q differentially regulate inflammatory gene induction by lupus immune complexes. J Immunol 188: 902–915. 22147767 10.4049/jimmunol.1102797 PMC3238790

[42] Pulliam L, Rempel H, Sun B, Abadjian L, Calosing C, et al (2011) A peripheral monocyte interferon phenotype in HIV infection correlates with a decrease in magnetic resonance spectroscopy metabolite concentrations. AIDS 25: 1721–1726. 21750421 10.1097/QAD.0b013e328349f022 PMC4120827

[43] Brenchley JM, Price DA, Douek DC (2006) HIV disease: fallout from a mucosal catastrophe? Nat Immunol 7: 235–239. 16482171 10.1038/ni1316

[44] Balagopal A, Philp FH, Astemborski J, Block TM, Mehta A, et al (2008) Human immunodeficiency virus-related microbial translocation and progression of hepatitis C. Gastroenterology. 135: 226–233. 10.1053/j.gastro.2008.03.022 PMC2644903 18457674

[45] Moir S, Chun TW, Fauci AS (2011) Pathogenic mechanisms of HIV disease. Annu Rev Pathol 6: 223–248. 21034222 10.1146/annurev-pathol-011110-130254

[46] Giorgi JV, Hultin LE, McKeating JA, Johnson TD, Owens B, et al (1999) Shorter survival in advanced human immunodeficiency virus type 1 infection is more closely associated with T lymphocyte activation than with plasma virus burden or virus chemokine coreceptor usage. J Infect Dis 179: 859–870. 10068581 10.1086/314660

[47] Bower M, Palmieri C, Dhillon T (2006) AIDS-related malignancies: changing epidemiology and the impact of highly active antiretroviral therapy. Curr Opin Infect Dis 19: 14–19. 16374212 10.1097/01.qco.0000200295.30285.13

[48] Battegay M, Nuesch R, Hirschel B, Kaufmann GR (2006) Immunological recovery and antiretroviral therapy in HIV-1 infection. Lancet Infect Dis 6: 280–287. 16631548 10.1016/S1473-3099(06)70463-7

[49] Steel A, John L, Shamji MH, Henderson DC, Gotch FM, et al (2008) CD38 expression on CD8 T cells has a weak association with CD4 T-cell recovery and is a poor marker of viral replication in HIV-1-infected patients on antiretroviral therapy. HIV Med 9: 118–125. 18257774 10.1111/j.1468-1293.2007.00528.x

[50] Heaton RK, Franklin DR, Ellis RJ, McCutchan JA, Letendre SL, et al (2011) HIV-associated neurocognitive disorders before and during the era of combination antiretroviral therapy: differences in rates, nature, and predictors. J Neurovirol 17: 3–16. 21174240 10.1007/s13365-010-0006-1 PMC3032197

[51] Nelson DR, Zeuzem S, Andreone P, Ferenci P, Herring R, et al (2012) Balapiravir plus peginterferon alfa-2a (40KD)/ribavirin in a randomized trial of hepatitis C genotype 1 patients. Ann Hepatol 11: 15–31. 22166557 PMC3739984

