# Supplementary material for: Monocyte Activation in HIV/HCV Coinfection Correlates with Cognitive Impairment
Source: PLoS One. 2013 Feb 21;8(2):e55776. doi: 10.1371/journal.pone.0055776 (PMC3578833; doi:10.1371/journal.pone.0055776)
Supplement: Table S1 — cDNA microarray probe intensities for monocyte genes induced in coinfected subjects (>2 fold change). (DOCX) [file pone.0055776.s001.docx]

| **Table S1. cDNA microarray probe intensities for monocyte genes induced in coinfected subjects (>2 fold change)** | | | | | | | |
| --- | --- | --- | --- | --- | --- | --- | --- |
| **Symbol** | **GenBank** | **C (HCV cohort)** | **HCV** | **HIV/HCV** | **C (HIV cohort)** | **HIV** | **HIV_UD_** |
| EOMES | NM_005442 | 14.7 (16.6) | 57.9 (14.9) | 151.7 (2.6) | 2.6 (17.6) | 4.4 (35.0) | 6.2 (13.6) |
| IFI27 | T47364 | 43.3 (1.9) | 145.2 (4.5) | 315.2 (5.6) | 25.6 (1.9) | 2973.6 (3.8) | 41.1 (1.7) |
| CD83 | NM_004233 | 76.4 (3.5) | 543.9 (3.8) | 495.0 (3.9) | 103.3 (2.5) | 91.6 (2.6) | 61.8 (1.9) |
| HBEGF | NM_001945 | 105.5 (23.4) | 521.1 (4.4) | 663.9 (3.7) | 176.9 (2.7) | 100.3 (2.1) | 67.9 (2.9) |
| FFAR2 | NM_005306 | 227.2 (21.6) | 1117.8 (2.1) | 1294.0 (2.1) | 286.0 (1.4) | 735.4 (1.6) | 254.2 (1.4) |
| SOCS3 | NM_003955 | 123.7 (19.2) | 547.9 (2.5) | 677.4 (2.9) | 128.6 (1.6) | 112.9 (2.2) | 73.0 (1.8) |
| ID1 | NM_181353 | 24.6 (15.6) | 68.3 (17.5) | 126.8 (2.8) | 15.2 (26.2) | 15.5 (12.0) | 12.3 (5.9) |
| SIGLEC1 | NM_023068 | 27.2 (13.6) | 77.2 (4.5) | 133.6 (3.0) | 25.9 (1.8) | 429.6 (2.5) | 32.3 (2.1) |
| G0S2 | NM_015714 | 1478.0 (4.8) | 4595.7 (4.4) | 7006.5 (2.9) | 1273.9 (3.2) | 660.8 (2.8) | 431.8 (3.0) |
| NFKB1 | AL598166 | 30.6 (15.8) | 131.1 (2.6) | 138.9 (2.4) | 116.6 (1.3) | 144.8 (1.5) | 114.5 (1.6) |
| CXCL2 | BF509029 | 428.4 (2.4) | 1273.8 (4.3) | 1827.9 (3.1) | 1141.1 (3.7) | 1126.6 (3.3) | 198.8 (34.7) |
| RGS1 | NM_002922 | 85.7 (4.6) | 210.5 (5.1) | 358.0 (4.0) | 260.8 (4.1) | 146.4 (7.4) | 111.7 (5.7) |
| ATF3 | NM_001030287 | 432.7 (3.4) | 2009.2 (3.3) | 1779.4 (3.6) | 460.8 (2.7) | 667.6 (2.1) | 219.1 (2.9) |
| TNFAIP3 | NM_006290 | 272.3 (3.2) | 1004.4 (2.7) | 985.2 (2.2) | 449.1 (1.9) | 511.7 (1.7) | 309.7 (1.4) |
| GPR183 | NM_004951 | 1051.9 (4.6) | 4337.3 (3.7) | 3292.7 (2.7) | 1991.2 (2.7) | 886.2 (2.5) | 656.6 (2.5) |
| ZNF331 | BX114572 | 69.1 (2.8) | 130.4 (1.8) | 216.0 (2.3) | 209.1 (1.8) | 91.8 (12.3) | 108.3 (2.1) |
| PFKFB3 | NM_004566 | 236.8 (3.5) | 581.8 (1.8) | 725.6 (2.0) | 229.0 (1.4) | 294.1 (1.3) | 218.6 (1.3) |
| GBP1P1 | BC013288 | 122.2 (1.7) | 335.3 (3.1) | 373.4 (2.3) | 75.2 (3.0) | 408.4 (1.7) | 106.9 (1.6) |
| PDE4B | NM_002600 | 366.2 (2.0) | 1024.7 (2.3) | 1118.8 (2.6) | 499.3 (1.6) | 430.6 (1.3) | 360.1 (1.3) |
| WARS | NM_004184 | 496.2 (3.3) | 1807.1 (1.4) | 1512.9 (1.8) | 1235.6 (1.4) | 1775.7 (1.4) | 1299.2 (1.6) |
| ETV7 | NM_016135 | 52.2 (2.8) | 102.9 (2.2) | 154.3 (2.7) | 27.8 (9.0) | 383.0 (2.0) | 87.9 (1.8) |
| CCL3 | NM_002983 | 1808.9 (4.5) | 4869.1 (3.7) | 5282.7 (2.8) | 3834.8 (2.8) | 2536.6 (3.7) | 988.5 (3.1) |
| CXCL11 | NM_005409 | 50.7 (2.9) | 121.1 (2.4) | 146.3 (2.4) | 54.4 (1.8) | 349.0 (1.8) | 72.2 (1.6) |
| NAMPT | AK023341 | 1129.3 (3.7) | 3292.9 (2.9) | 3232.6 (2.5) | 2431.7 (2.2) | 1923.3 (2.4) | 687.1 (3.0) |
| PTGS2 | NM_000963 | 815.9 (3.4) | 2560.7 (4.6) | 2312.6 (2.9) | 1686.1 (2.5) | 859.0 (2.7) | 472.9 (3.3) |
| IL10 | NM_000572 | 282.1 (2.2) | 614.7 (3.2) | 790.1 (3.8) | 318.5 (2.3) | 376.7 (2.0) | 193.4 (1.9) |
| CCL4 | CD522041 | 803.9 (2.3) | 2447.4 (3.5) | 2240.3 (2.6) | 1047.8 (2.9) | 1252.3 (3.4) | 626.6 (2.0) |
| THBS1 | H99504 | 568.3 (3.3) | 2024.3 (4.2) | 1573.9 (4.1) | 731.6 (3.0) | 139.3 (21.3) | 230.9 (3.8) |
| GRASP | NM_181711 | 538.8 (1.9) | 964.4 (2.9) | 1486.8 (2.6) | 399.8 (2.1) | 329.5 (1.5) | 257.4 (2.1) |
| EREG | NM_001432 | 141.9 (3.2) | 402.6 (3.7) | 390.6 (3.3) | 185.2 (2.5) | 124.5 (2.3) | 84.9 (2.4) |
| FCAR | NM_002000 | 203.6 (3.8) | 609.7 (2.1) | 558.2 (2.1) | 349.8 (1.6) | 366.1 (1.6) | 268.3 (1.4) |
| GBP1 | NM_002053 | 833.3 (2.1) | 1571.6 (1.8) | 2280.2 (1.8) | 939.4 (1.7) | 3428.9 (1.6) | 1436.0 (1.5) |
| NFIL3 | NM_005384 | 202.3 (3.0) | 515.4 (2.4) | 553.1 (2.0) | 223.9 (1.6) | 238.9 (1.5) | 153.2 (1.5) |
| PHLDA2 | NM_003311 | 180.8 (1.9) | 363.0 (2.9) | 482.6 (2.5) | 89.2 (1.3) | 129.0 (1.7) | 87.8 (1.5) |
| PTX3 | NM_002852 | 65.4 (2.4) | 197.2 (3.1) | 173.7 (2.3) | 92.7 (1.4) | 111.3 (1.6) | 70.2 (1.3) |
| C15orf48 | NM_032413 | 111.3 (1.4) | 225.3 (3.5) | 295.3 (3.0) | 79.8 (1.5) | 141.0 (1.8) | 76.7 (1.5) |
| VRK2 | NM_006296 | 93.9 (3.0) | 263.7 (1.5) | 248.2 (1.9) | 162.6 (1.4) | 268.2 (1.3) | 199.6 (1.2) |
| RSAD2 | NM_080657 | 123.9 (2.2) | 313.7 (2.1) | 322.1 (2.5) | 201.3 (1.4) | 1407.6 (2.1) | 214.8 (1.5) |
| GBP2 | NM_004120 | 993.1 (2.1) | 2331.1 (1.5) | 2579.6 (1.6) | 2042.2 (1.4) | 3073.0 (1.3) | 2222.2 (1.4) |
| SIK1 | NM_173354 | 502.3 (2.4) | 958.0 (2.8) | 1270.3 (2.4) | 584.4 (2.5) | 475.0 (2.0) | 306.0 (2.7) |
| CXCL10 | NM_001565 | 657.0 (2.1) | 1367.6 (1.9) | 1653.8 (2.0) | 690.5 (1.5) | 2849.9 (1.5) | 987.5 (1.5) |
| SERPING1 | NM_000062 | 541.3 (2.2) | 692.1 (2.2) | 1354.5 (2.5) | 237.1 (1.6) | 2005.8 (2.0) | 369.9 (1.6) |
| ARL4C | BM918203 | 178.0 (2.7) | 606.3 (2.0) | 445.1 (2.5) | 279.9 (2.2) | 92.1 (11.7) | 147.0 (2.3) |
| NFKB2 | NM_002502 | 299.7 (2.4) | 782.0 (1.6) | 738.9 (1.7) | 505.8 (1.3) | 489.8 (1.4) | 397.7 (1.4) |
| UBE2D3 | AA255716 | 270.6 (2.2) | 694.9 (2.0) | 657.1 (1.6) | 1029.0 (1.4) | 928.2 (1.4) | 667.3 (1.4) |
| APOBEC3A | NM_145699 | 2017.2 (2.7) | 4626.7 (1.6) | 4857.7 (1.6) | 2076.6 (1.4) | 6430.3 (1.4) | 1973.6 (1.4) |
| CD69 | NM_001781 | 131.8 (1.9) | 289.1 (1.6) | 316.8 (2.3) | 146.3 (1.8) | 158.3 (1.8) | 108.0 (1.6) |
| NFKBIZ | NM_001005474 | 2287.8 (2.3) | 5715.5 (1.8) | 5476.3 (1.4) | 5826.4 (1.4) | 5794.8 (1.5) | 3950.2 (1.4) |
| LGALS9 | BF347161 | 456.4 (3.1) | 1542.2 (2.2) | 1083.9 (2.8) | 704.6 (1.7) | 1004.5 (1.6) | 774.1 (1.5) |
| STAT1 | NM_007315 | 1313.5 (1.7) | 2472.8 (1.5) | 3107.7 (1.5) | 2425.2 (1.4) | 5087.0 (1.2) | 2979.7 (1.2) |
| PNPLA2 | X56789 | 140.2 (3.6) | 520.6 (1.7) | 329.2 (2.4) | 297.5 (1.4) | 204.4 (1.4) | 328.9 (1.3) |
| FPR2 | NM_001005738 | 469.6 (3.0) | 1203.1 (1.7) | 1102.2 (1.7) | 922.2 (1.4) | 1517.1 (1.5) | 828.9 (1.2) |
| C9orf72 | NM_145005 | 405.3 (2.3) | 894.2 (1.8) | 949.5 (1.7) | 671.9 (1.6) | 780.6 (1.5) | 559.0 (1.4) |
| DDIT3 | H25717 | 173.4 (2.2) | 452.5 (2.3) | 403.9 (1.9) | 193.2 (1.6) | 148.9 (1.6) | 108.4 (1.4) |
| C1QC | NM_172369 | 82.6 (1.9) | 118.8 (2.4) | 190.6 (2.4) | 39.0 (3.1) | 183.6 (2.5) | 73.4 (1.6) |
| HES4 | NM_021170 | 753.6 (1.8) | 1496.8 (2.6) | 1738.2 (2.2) | 460.6 (1.5) | 1062.4 (1.5) | 397.4 (1.6) |
| ZNF394 | NM_032164 | 393.9 (2.9) | 991.2 (1.6) | 905.0 (1.5) | 511.1 (1.2) | 564.6 (1.2) | 473.9 (1.2) |
| IFI44L | NM_006820 | 1268.6 (1.9) | 1869.1 (2.5) | 2912.9 (2.5) | 1169.3 (1.5) | 7396.4 (1.6) | 1363.1 (2.0) |
| STK17B | NM_004226 | 202.8 (3.2) | 470.7 (2.3) | 463.3 (1.9) | 242.8 (1.9) | 241.8 (1.3) | 205.9 (1.4) |
| GABRD | NM_000815 | 73.5 (3.5) | 207.0 (1.3) | 167.7 (2.2) | 148.4 (1.3) | 88.1 (11.0) | 119.5 (1.2) |
| PXK | BF509422 | 250.5 (1.7) | 524.8 (2.3) | 565.9 (2.1) | 145.4 (1.3) | 142.7 (1.5) | 139.1 (1.4) |
| PRDM1 | NM_001198 | 214.7 (1.6) | 412.7 (2.0) | 484.9 (1.9) | 241.0 (1.3) | 284.8 (1.5) | 213.9 (1.4) |
| DGKA | NM_001345 | 63.2 (3.7) | 168.0 (1.8) | 142.6 (2.5) | 106.8 (1.4) | 101.0 (1.7) | 93.7 (1.8) |
| RIPK2 | NM_003821 | 1258.5 (1.9) | 2515.7 (1.8) | 2840.0 (1.7) | 1461.8 (1.4) | 1425.1 (1.4) | 1144.3 (1.2) |
| MX1 | BG055292 | 175.5 (1.5) | 373.9 (2.0) | 395.7 (2.0) | 235.9 (1.3) | 777.3 (1.6) | 253.9 (1.5) |
| TMEM176B | NM_014020 | 2283.8 (2.7) | 5941.5 (2.5) | 5139.3 (2.9) | 4802.9 (2.9) | 2994.5 (2.8) | 7890.1 (2.1) |
| MXD1 | L06895 | 110.0 (1.8) | 239.0 (2.1) | 246.5 (2.3) | 114.8 (1.5) | 146.3 (1.6) | 81.8 (1.2) |
| ATP1B3 | N32570 | 426.0 (4.2) | 991.4 (1.8) | 945.7 (1.9) | 1106.1 (1.7) | 1031.8 (1.8) | 823.1 (1.4) |
| P2RX7 | NM_002562 | 87.6 (3.0) | 173.2 (1.6) | 193.2 (1.9) | 212.1 (1.5) | 273.0 (1.5) | 228.0 (1.7) |
| CD33 | NM_001772 | 1323.3 (2.7) | 2897.0 (1.7) | 2905.4 (1.7) | 3677.3 (1.5) | 3338.5 (1.3) | 2778.9 (1.5) |
| EIF2S3 | BM807150 | 469.1 (2.8) | 1378.6 (1.9) | 1028.1 (2.2) | 1740.5 (1.7) | 1294.3 (1.6) | 1704.5 (1.5) |
| GNPDA1 | NM_005471 | 71.2 (3.3) | 174.6 (1.8) | 155.7 (2.3) | 144.0 (1.5) | 152.4 (1.6) | 133.8 (1.6) |
| LAP3 | AA478224 | 1900.0 (2.0) | 3293.6 (1.5) | 4151.0 (1.4) | 1599.7 (1.4) | 4324.6 (1.4) | 2172.2 (1.3) |
| GMPS | NM_003875 | 113.5 (3.8) | 286.4 (1.5) | 247.1 (1.8) | 306.8 (1.5) | 158.4 (12.9) | 279.5 (1.4) |
| OAF | NM_178507 | 239.0 (1.3) | 440.8 (1.4) | 518.7 (1.7) | 192.6 (1.3) | 207.9 (1.5) | 194.3 (1.5) |
| PPT1 | NM_000310 | 956.7 (3.2) | 3187.6 (1.4) | 2071.6 (2.3) | 2730.9 (1.3) | 2756.4 (1.4) | 2665.3 (1.3) |
| LGALS3BP | NM_005567 | 842.2 (1.6) | 929.7 (2.1) | 1814.4 (2.3) | 602.3 (1.6) | 4181.3 (1.9) | 1082.2 (1.4) |
| PLSCR1 | NM_021105 | 1003.2 (2.9) | 2248.1 (1.5) | 2153.0 (1.8) | 1479.9 (1.3) | 3106.4 (1.5) | 1591.9 (1.3) |
| LTA4H | BP377500 | 954.8 (3.6) | 2060.2 (1.6) | 2047.0 (2.1) | 2578.6 (1.3) | 1600.7 (1.5) | 2675.9 (1.3) |
| ARL5B | AF494061 | 74.1 (1.7) | 123.0 (3.0) | 158.7 (2.5) | 67.6 (1.4) | 76.4 (1.5) | 50.0 (1.5) |
| TPMT | U12387 | 146.7 (3.2) | 361.1 (1.6) | 314.0 (1.9) | 273.5 (1.6) | 394.3 (1.4) | 277.5 (1.2) |
| XAF1 | NM_017523 | 1401.6 (1.8) | 2473.1 (2.0) | 2978.5 (2.0) | 2109.4 (1.5) | 6729.5 (1.6) | 2720.8 (1.3) |
| BIRC3 | NM_001165 | 152.5 (1.7) | 337.7 (1.7) | 324.1 (1.5) | 350.2 (1.4) | 510.4 (1.4) | 361.1 (1.3) |
| GABARAPL1 | NM_031412 | 860.9 (1.9) | 1576.2 (2.1) | 1819.6 (2.2) | 806.8 (1.5) | 741.0 (1.4) | 652.7 (1.5) |
| FAM198B | AY358785 | 225.4 (1.8) | 478.3 (2.5) | 476.3 (2.4) | 723.1 (1.7) | 666.7 (1.5) | 740.0 (1.6) |
| HNRNPH2 | NM_019597 | 269.7 (3.0) | 708.0 (1.7) | 568.4 (2.4) | 357.0 (1.9) | 306.5 (2.0) | 287.6 (1.8) |
| SNX2 | NM_003100 | 1214.6 (2.6) | 3003.4 (1.6) | 2553.1 (2.0) | 2976.1 (1.5) | 1589.6 (21.1) | 2555.4 (1.4) |
| SQRDL | NM_021199 | 876.8 (3.1) | 2289.4 (1.3) | 1842.2 (1.7) | 1924.5 (1.4) | 2351.0 (1.4) | 1851.0 (1.3) |
| SERINC1 | NM_020755 | 726.5 (3.0) | 2010.8 (1.7) | 1524.1 (2.3) | 1940.1 (1.5) | 2080.3 (1.4) | 1777.6 (1.4) |
| DMXL2 | NM_015263 | 127.6 (2.7) | 270.8 (1.5) | 267.1 (1.9) | 473.2 (1.4) | 546.7 (1.4) | 445.4 (1.3) |
| EEF1A1 | BU902820 | 13104.6 (2.4) | 31998.0 (1.8) | 27323.0 (1.9) | 24838.6 (2.0) | 23200.1 (1.8) | 28234.0 (2.0) |
| ADSS | NM_001126 | 339.5 (2.5) | 788.6 (1.6) | 705.4 (1.9) | 781.8 (1.8) | 760.8 (1.5) | 623.6 (1.4) |
| LYN | NM_002350 | 1751.7 (2.5) | 3607.0 (1.8) | 3633.5 (1.7) | 2935.2 (1.3) | 3563.8 (1.3) | 2997.6 (1.3) |
| OAS1 | NM_002534 | 2099.5 (2.4) | 4714.1 (1.6) | 4352.8 (1.9) | 4248.5 (1.4) | 7503.4 (1.6) | 3996.7 (1.5) |
| DAZAP2 | NM_014764 | 3470.1 (2.3) | 9277.9 (1.5) | 7188.2 (2.0) | 5656.9 (1.5) | 5437.5 (1.5) | 5022.7 (1.5) |
| RBBP4 | NM_005610 | 498.9 (2.4) | 1181.2 (1.8) | 1031.6 (2.1) | 1303.8 (1.6) | 1265.0 (1.5) | 1226.2 (1.5) |
| CCNG1 | NM_004060 | 245.9 (2.5) | 638.6 (1.9) | 506.4 (2.2) | 598.0 (1.6) | 537.1 (1.5) | 564.6 (1.3) |
| NEAT1 | AF001893 | 411.0 (2.9) | 851.3 (2.1) | 843.3 (1.8) | 1609.1 (1.6) | 1599.1 (1.4) | 1254.7 (1.7) |
| IFI44 | NM_006417 | 1148.9 (2.1) | 2254.1 (2.0) | 2357.7 (2.3) | 1177.7 (1.3) | 4528.1 (1.8) | 1439.7 (1.4) |
| PTPRC | NM_002838 | 1586.0 (2.4) | 3590.3 (1.5) | 3246.5 (1.7) | 4655.2 (1.3) | 5540.4 (1.2) | 5019.3 (1.2) |
| OAT | NM_000274 | 283.5 (2.6) | 779.7 (1.7) | 577.4 (2.4) | 564.8 (1.6) | 500.4 (1.6) | 459.7 (1.5) |
| TRA2B | NM_004593 | 1137.0 (2.7) | 2710.0 (1.5) | 2312.0 (1.4) | 1210.4 (1.4) | 1149.7 (1.5) | 1154.4 (1.4) |
| DUSP1 | NM_004417 | 3592.1 (3.4) | 7839.1 (2.1) | 7294.6 (1.5) | 6724.5 (1.7) | 5289.9 (1.5) | 4416.7 (1.6) |
| SNX1 | T33691 | 285.8 (2.9) | 660.4 (1.5) | 579.9 (2.2) | 956.7 (1.4) | 992.7 (1.4) | 867.1 (1.4) |
| FGL2 | BG400482 | 3296.5 (2.8) | 7429.9 (1.8) | 6688.1 (1.9) | 8848.9 (1.3) | 7574.7 (1.7) | 7321.3 (1.4) |
| EPSTI1 | NM_001002264 | 574.3 (1.9) | 788.1 (1.7) | 1161.6 (2.0) | 448.1 (1.4) | 2137.3 (1.7) | 629.6 (1.4) |
| GOLPH3 | NM_022130 | 822.0 (3.6) | 1683.0 (1.2) | 1657.9 (1.4) | 2319.8 (1.3) | 2466.4 (1.2) | 2251.8 (1.1) |
| SELT | BM710895 | 2049.2 (3.3) | 5047.8 (1.4) | 4132.6 (1.9) | 4623.9 (1.4) | 4498.7 (1.3) | 4078.5 (1.2) |
| AOAH | NM_001637 | 858.6 (1.9) | 1949.5 (1.4) | 1730.0 (1.8) | 1448.1 (1.5) | 1504.1 (1.5) | 1531.0 (1.6) |
| CSNK1A1 | L37042 | 796.1 (2.0) | 1841.0 (1.4) | 1603.1 (1.7) | 1582.3 (1.4) | 1855.1 (1.3) | 1481.8 (1.3) |
| ID2 | NM_002166 | 1212.0 (1.7) | 2247.0 (1.5) | 2424.8 (1.5) | 1131.1 (1.4) | 1245.4 (1.4) | 1272.1 (1.4) |
| **Mean (± standard error)** | |  |  |  |  |  |  |
